# Fetal growth is driven by a bimodal fetal beta cell response and reshaped by a ketogenic diet in a mouse model of maternal diabetes

**DOI:** 10.1007/s00125-026-06771-w

**Published:** 2026-06-09

**Authors:** Omer Cohenshtam, Amnon Zung, Amy Yu, Hadar King, Polina Kotova, Ofer Gover, Itia Magenheim Samuel, Sarit Helman, Michael D. Walker, Danny Ben-Zvi, Naama Kanarek, Aharon Helman

**Affiliations:** 1 Department of Biochemistry, Food Science and Nutrition, Robert H. Smith Faculty of Agriculture, Food and Environment, Hebrew University of Jerusalem, Rehovot, Israel; 2 Pediatric Endocrinology Unit, Kaplan Medical Center, Rehovot, Israel; 3 Department of Pathology, Boston Children’s Hospital, Boston, MA USA; 4 Graduate Program in Biological and Biomedical Sciences, Harvard Medical School, Boston, MA USA; 5 Department of Biomolecular Sciences, Weizmann Institute of Science, Rehovot, Israel; 6 Department of Developmental Biology and Cancer Research, Institute for Medical Research Israel–Canada, The Hebrew University of Jerusalem, Jerusalem, Israel; 7 Department of Obstetrics & Gynecology, Shaare Zedek Medical Center, Faculty of Medicine, Hebrew University of Jerusalem, Jerusalem, Israel; 8 Broad Institute of Massachusetts Institute of Technology (MIT) and Harvard, Cambridge, MA USA; 9 Harvard Medical School, Boston, MA USA

**Keywords:** Beta cell maturation, Fetal beta cell, Fetal growth, Insulin secretion, IUGR, Ketogenic diet, Macrosomia, Maternal diabetes, mTORC1, Pregnancy

## Abstract

**Aims/hypothesis:**

Maternal diabetes confers two opposing risks to fetal growth, resulting in macrosomia in mild cases and intrauterine growth restriction (IUGR) in severe cases. The mechanisms governing these divergent responses are poorly understood, given the intimate regulation of insulin by glucose and insulin’s fetal growth-promoting effects. We hypothesised that the degree of maternal hyperglycaemia dictates a bimodal pattern of fetal insulin secretion that determines fetal growth, and that use of a ketogenic diet (KD) as a nutritional intervention could modify this outcome.

**Methods:**

We used the Insulin-rtTA;TET-DTA mouse model to induce preconception diabetes. Dams were stratified based on maternal blood glucose, namely non-diabetes (glucose <9.6 mmol/l), mild diabetes (glucose range 9.6–16.7 mmol/l) or severe diabetes (glucose >16.7 mmol/l), and maintained on either a normal diet or a KD. We assessed fetal growth and plasma C-peptide, performed islet functional assays ex vivo, and characterised changes in plasma metabolites. Fetal pancreases were analysed by immunohistochemistry for beta cell area, proliferation, maturation and mechanistic target of rapamycin complex 1 (mTORC1) activity.

**Results:**

Mild maternal diabetes induced fetal macrosomia, driven by beta cell hyperplasia, hyperinsulinaemia and premature beta cell functional maturation, as reflected by glucose-stimulated insulin secretion and upregulated MafA expression. This was associated with strong activation of the mTORC1 pathway. In contrast, severe diabetes caused IUGR associated with reduced beta cell mass and profound functional impairment. The KD had divergent effects: it normalised fetal growth in the mild diabetes group by preventing beta cell proliferation and premature maturation, thereby reducing insulin secretion, but failed to rescue IUGR in the severe diabetes group, despite partially restoring beta cell function. Notably, the KD uncoupled the positive correlation between fetal insulin and body weight, revealing a primary, insulin-independent, growth-restrictive effect.

**Conclusions/interpretation:**

Fetal growth in a mouse model of diabetes in pregnancy is governed by a bimodal beta cell response to the maternal glycaemic environment, orchestrated at the molecular level by the mTORC1 pathway. A KD can prevent diabetes-derived macrosomia by reducing beta cell stimulation and through insulin-independent mechanisms, but cannot reverse IUGR, warranting further studies of its role in diabetes during pregnancy.

**Graphical Abstract:**

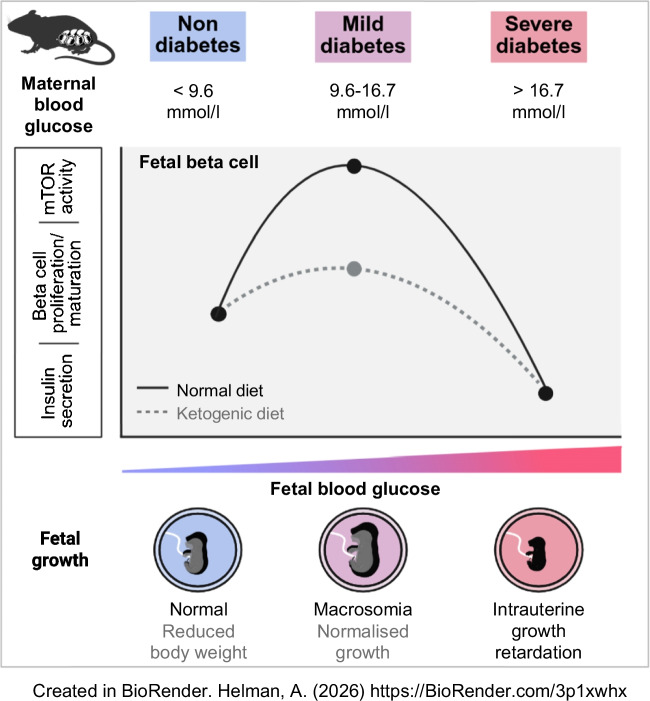

**Supplementary Information:**

The online version of this article (10.1007/s00125-026-06771-w) contains peer-reviewed but unedited supplementary material.


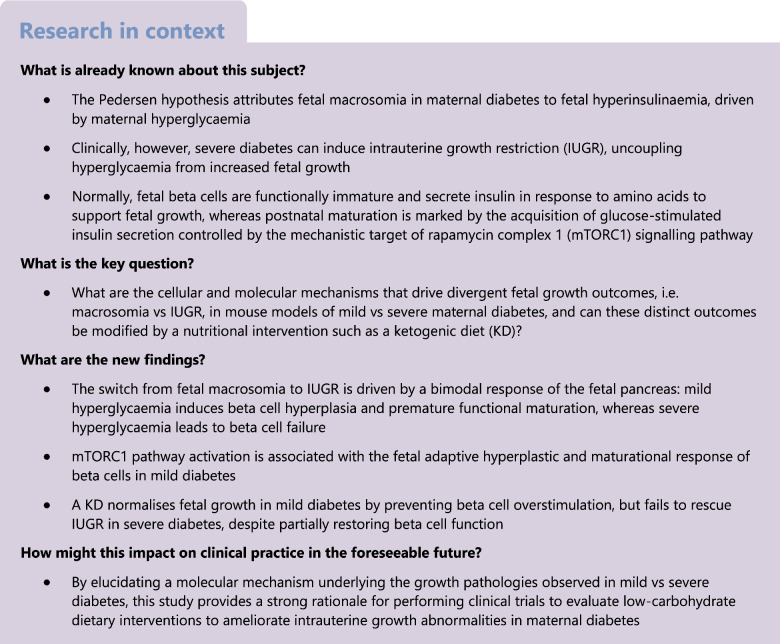


## Introduction

The prevalence of preconception diabetes has doubled over the last 20 years, complicating approximately 1% of pregnancies worldwide and conferring substantial risks to both mother and fetus [1]. These risks include spontaneous abortion, congenital malformations, pre-eclampsia, fetal demise, macrosomia and postnatal hypoglycaemia, hyperbilirubinaemia and respiratory distress syndrome [1]. The currently accepted explanation for fetal macrosomia is the Pedersen hypothesis, which posits that maternal hyperglycaemia leads to fetal hyperinsulinaemia and subsequent intrauterine overgrowth [2]. In apparent contradiction to this hypothesis, severe diabetes can paradoxically result in intrauterine growth restriction (IUGR) rather than macrosomia [3, 4]. The mechanisms governing this switch from fetal overgrowth to growth failure are poorly understood. This complexity is compounded by the fact that fetal beta cells are functionally immature and respond primarily to amino acids (AA) rather than glucose: the switch to a mature, glucose-stimulated insulin secretion (GSIS) pattern emerges only after birth [5]. Nevertheless, several studies have shown that maternal hyperglycaemia during pregnancy increases pancreatic insulin content and secretion in embryos [6], and that fetal beta cells cultured in high-glucose medium acquire glucose responsiveness ex vivo [7, 8].

Resolving the paradoxical uncoupling of hyperglycaemia from intrauterine growth requires determination of the molecular pathways that regulate fetal beta cell fate in response to the severity of maternal diabetes. The mechanistic target of rapamycin complex 1 (mTORC1) pathway is a prime candidate for this role. As a central regulator of cell growth and a key nutrient sensor, mTORC1 is critical for islet proliferation and function during the perinatal period [9, 10]. We previously showed that a postnatal nutritional shift drives mTORC1 to convert beta cells to a glucose-responsive state [5]. In the present study, using a mouse model, we aimed to determine whether the degree of in utero hyperglycaemia modifies the mTORC1 pathway, driving adaptive beta cell proliferation and maturation in mild diabetes, but causing beta cell exhaustion and failure under the stress of severe hyperglycaemia. We further aimed to evaluate a glucose-restricted nutritional intervention during pregnancy as a putative therapeutic option that may reshape intrauterine growth in response to maternal hyperglycaemia. While strict carbohydrate restriction, such as a ketogenic diet (KD), is not recommended during pregnancy [1, 11–15], its robust effect on systemic metabolism makes it a valuable tool for mechanistic investigation. We hypothesised that, by providing an alternative fuel source and dramatically reducing the glucose load, a KD could protect fetal beta cells from pathological overstimulation and glucotoxicity, potentially reducing the risk of impaired fetal growth.

## Methods

### Mice

The study was approved by the ethics committee of the Hebrew University of Jerusalem (MD-22-168544). We established diabetes thresholds based on 154 non-fasting blood glucose (BG) measurements from 40 healthy female Hsd:ICR (CD-1) (Harlan Laboratories, Israel; www.inotiv.com/research-model/hsd-icr-cd-1) aged 9.1 ± 5.6 weeks. All animals were housed in a specific pathogen-free (SPF) barrier facility under a controlled 12 h light/dark cycle. Environmental enrichment was provided in all cages. The experimental groups received either a normal diet (ND) or a KD ad libitum. The mean BG was 6.88 ± 1.39 mmol/l, and we defined diabetes as two standard deviations above this mean, namely >9.6 mmol/l. We further divided the diabetes definition into mild (9.6−16.7 mmol/l) and severe (>16.7 mmol/l) categories for use in stratifying the dams throughout the study.

Preconception diabetes was induced in double-transgenic Insulin-rtTA;TET-DTA female mice (Tg[Ins2-rtTA]2Efr Tg[teto-DTA]1Gfi/J) from The Jackson Laboratory (USA; www.jax.org/strain/008755; generously donated by Y. Dor, Department of Molecular Biology, The Hebrew University of Jerusalem, Israel), as previously described [16]. Specifically, doxycycline was administered to induce variable degrees of beta cell ablation and consequently variable degrees of hyperglycaemia [17, 18]. Single-transgenic female mice (Tg[Ins2-rtTA]2Efr or Tg[tetO-DTA]1Gfi/J; The Jackson Laboratory; www.jax.org/strain/008250 and www.jax.org/strain/008168, respectively) were used as the non-diabetes group (group with BG <9.6 mmol/l). Non-fasting BG measurements were taken twice weekly between 11:00 and 14:00 hours throughout the study, using a FreeStyle glucometer (Abbott). For diabetes induction, doxycycline (2 mg/ml doxycycline, 4% w/v sucrose) was administered in the drinking water at a mean age of 6.0 ± 2.9 weeks for 7 days. Similarly, 4% w/v sucrose in the drinking water was administered to mice in the non-diabetes group. Female mice were then mated with Hsd:ICR (CD-1) (Harlan Laboratories; www.inotiv.com/research-model/hsd-icr-cd-1) male mice at a mean age of 10.7 ± 3.5 weeks. At day E18.5 of pregnancy, mice were fasted for 3 h and then killed under general anaesthesia comprising 60–120 µl ketamine (1 g/10 ml) and 60–120 µl xylazine (20 mg/ml) per mouse, and maternal BG and body weight (BW) were measured. Embryo BW, body length (BL), placental weight and BG levels were documented. Blood samples were drawn from the dams under general anaesthesia just prior to euthanasia, while embryonic blood was collected and pooled per litter. All blood samples were maintained on ice in heparinised tubes until centrifugation at 10,000 *g* for 20 min at 4°C. The extracted plasma samples were stored at −80°C for C-peptide and β-hydroxybutyrate measurements and metabolic profiling. Seven dams with litters of fewer than eight embryos were excluded from the study: two from the non-diabetes group, three from the mild diabetes group and two from the severe diabetes group. Masking was not carried out for these experiments.

For further details, including details of housing and husbandry, please see electronic supplementary material (ESM) Methods.

### Insulin/C-peptide measurements

Both insulin and C-peptide were measured using ELISA kits (Crystal Chem) according to the manufacturer’s instructions. For further details, please see ESM Methods.

### Fetal islet preparation

Following maternal euthanasia (in a separate process), embryos (E18.5) were killed, and the pancreas of each embryo was surgically removed under the microscope and transferred to a plate containing HBSS (Ca^2+^/Mg^2+^) (Biological Industries). Five pancreases were then pooled in 2 ml Eppendorf tubes kept on ice, and the tubes were centrifuged (200 *g* for 2 min at room temperature). After removing the supernatant, 600 µl collagenase solution (Roche; diluted in 100 mg/ml HBSS) was added, and the tubes were incubated for 15 min in a 37°C water bath, with inversion every 30 s. Collagenase digestion was stopped by adding 1 ml RPMI (11 mmol/l glucose, 10% FBS, 1% penicillin/streptomycin and 1% glutamine). After centrifugation (200 *g* for 2 min at room temperature), the supernatant was removed, RPMI was added, and the suspension was transferred to a 15 ml tube containing 2 ml Histopaque 1077 (Sigma-Aldrich) for purification. The tubes were then centrifuged (600 *g*, 20 min, 4°C) with very slow acceleration and braking, and the upper and interphase layers were transferred to plates containing RPMI. The pellet in the tubes was resuspended in 1 ml HBSS (+Ca^2+^/Mg^2+^), and subjected again to collagenase digestion and Histopaque purification, except that the incubation in the second round was limited to 6 min at 37°C. Isolated islets from both steps were combined and cultured overnight at 37°C, 5% CO_2_ in RPMI.

### Ex vivo GSIS in fetal islets

Three experimental conditions were tested: 2.8 mmol/l glucose without AA, 2.8 mmol/l glucose with AA, and 16.7 mmol/l glucose with AA. The experimental conditions comprised KRBH (119 mmol/l NaCl, 20 mmol/l HEPES, 4.6 mmol/l KCl, 1 mmol/l MgSO_4_·7H_2_O, 0.15 mmol/l Na_2_PO_4_, 0.4 mmol/l KH_2_PO_4_ and 5 mmol/l NaHCO_3_), 0.05% v/v BSA, 2 mmol/l CaCl_2_ and the designated glucose concentrations. After overnight incubation in RPMI, islets were individually picked under a dissecting microscope, and transferred to 0.5 ml tubes containing RPMI, with nine islets per tube. The tubes were centrifuged (200 *g* for 2 min at room temperature) and the supernatant was discarded. Subsequently, islets were incubated with 2.8 mmol/l glucose without AA for 30 min. The tubes were then centrifuged (200 *g* for 2 min at room temperature), the supernatant was discarded, and islets were incubated under each of the three experimental conditions for 60 min. Each condition was tested in 5–7 biological replicates. Finally, the tubes were centrifuged (200* g* for 2 min at room temperature), and the supernatant was collected and stored at −80°C.

### Immunohistochemistry

Fetal pancreases were surgically removed and immediately immersed in 4% paraformaldehyde solution for overnight fixation at 4°C, then processed and embedded in paraffin blocks. Pancreas tissue slices were used for immunohistochemical staining to detect insulin, phosphorylated ribosomal protein S6 (p-S6), MafA and Ki-67. Each islet was manually delineated to measure the percentage of beta cell area relative to total tissue area, and to quantify the intensities of MafA, Ki-67 and p-S6. For further details, please see ESM Methods.

### Ketogenic diet

The KD used (Envigo, TD96355) is characterised by a very high fat content and very low carbohydrate content. A comparison of the nutrients and caloric composition between the ND and the KD is provided in Table 1.

**Table 1:** Macronutrient content of the ND and the KD

	Energy density^a^, kcal/g	Protein, % by weight (% by energy)	Carbohydrates, % by weight (% by energy)	Fat, % by weight (% by energy)
ND	3.0	24.3 (32)	40.2 (54)	4.7 (14)
KD	6.7	15.3 (9.2)	0.5 (0.3)	67.4 (90.5)

^a^Energy density is a calculated estimate of metabolisable energy based on the known caloric content of the macronutrients

### β-Hydroxybutyrate quantification

β-Hydroxybutyrate concentrations were quantified using a FreeStyle Optium Neo system (Abbott, 7869001). For further details, please see ESM Methods.

### LC-MS polar metabolite analysis

Samples were analysed using Vanquish Flex UHPLC paired with a Q-Exactive Orbitrap system (Thermo Fisher Scientific). Polar metabolites were detected using a 150 × 2.1 mm ZIC-pHILIC column (5 μm particle size; EMD Millipore). For further details, please see ESM Methods.

### Metabolomics data analysis

Polar metabolites were quantified relative to an in-house library of chemical standards using Trace Finder version 4.1 (Thermo Fisher Scientific). Data normalisations were performed using both the averaged peak area of the internal standards for detection of technical errors, and the averaged detected abundance of high-quality peaks per sample for detection of variation in the amount or volume of biological material. For further details, please see ESM Methods.

### Statistical analysis

Statistical analyses were performed using GraphPad Prism software version 9.0.0 (GraphPad, Boston, MA, USA). Comparisons between two groups were performed using an unpaired Student’s *t* test. Multiple comparisons were conducted using one-way analysis of variance (ANOVA) for comparisons of diabetes groups alone, whereas two-way ANOVA with Tukey’s post hoc test was used for comparisons incorporating dietary interventions. Linear regression was employed to analyse associations between variables, with Pearson correlation being used to determine the strength of the linear relationship. Data are presented as mean ± SD. A *p* value of <0.05 was considered statistically significant.

## Results

### Maternal diabetes severity dictates fetal growth in a bimodal manner

**Fig. 1:**
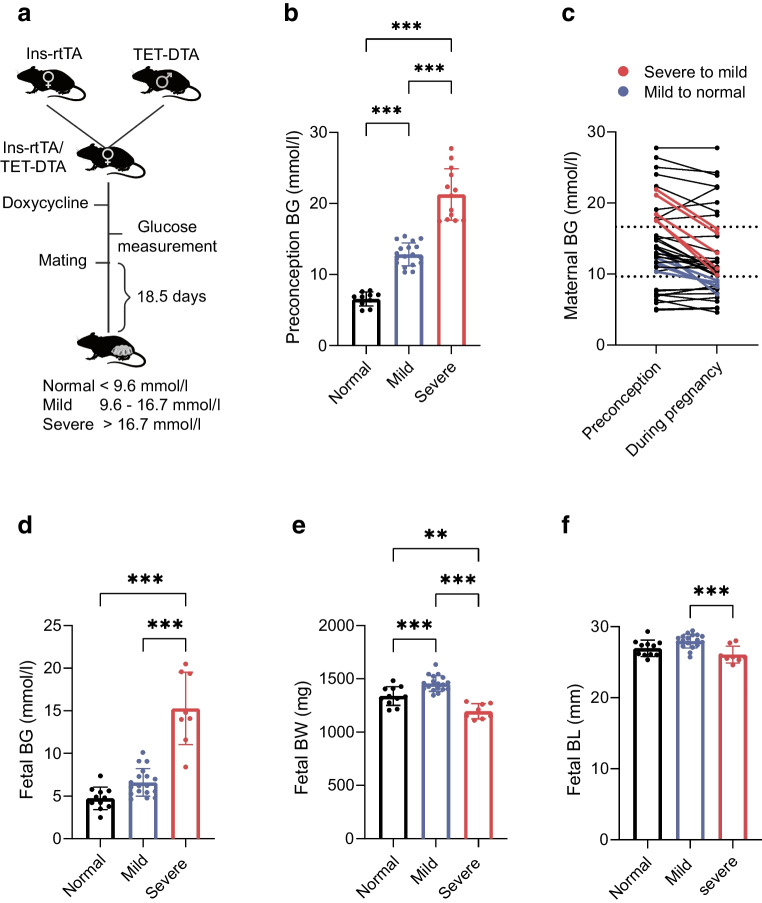
Maternal diabetes severity dictates fetal growth in a bimodal manner. (**a**) Schematic experimental representation of induction of preconception diabetes in Insulin-rtTA;TET-DTA mice. Created in BioRender. Helman, A. (2026) https://BioRender.com/3p1xwhx. (**b**) Mean BG for dams before pregnancy based on twice-weekly measurements in the non-diabetes (normal; *n*=11), mild diabetes (*n*=18) and severe diabetes (*n*=13) groups. BG data are missing for *n*=1 dam in the non-diabetes group. (**c**) Maternal BG dynamics between preconception doxycycline-induced diabetes and pregnancy. Red lines indicate dams that shifted from the severe to the mild diabetes range (*n*=5) and blue lines indicates dams that reverted from mild diabetes to normoglycaemia (*n*=5). Black lines indicate dams whose BG remained steady prior to and during pregnancy. (**d**–**f**) Fetal BG (**d**), fetal BW (**e**) and fetal BL (**f**) at E18.5 in the non-diabetes (normal; *n*=11), mild diabetes (*n*=18) and severe diabetes (*n*=8) groups. Five litters of dams with a BG decrease from the severe to the mild diabetes range were excluded. Each point represents the mean value for the litter. Data are presented as mean ± SD (**b**, **d**–**f**) or mean only (**c**) and analysed by one-way ANOVA; **p<0.01, ***p<0.001

Following doxycycline administration and diabetes induction, we stratified 42 dams aged 13 ± 3.8 weeks at delivery into three groups based on preconception BG levels: non-diabetes (*n*=11), mild diabetes (*n*=18) and severe diabetes (*n*=13) (Fig. 1a). As expected, maternal BG levels were significantly different between the groups both before and during pregnancy (Fig. 1b and ESM Fig. 1a). These differences were also maintained when maternal BG levels were grouped by the trimesters of pregnancy (ESM Fig. 1b). The glycaemic status in some dams improved during pregnancy: the mean BG in five mice from the severe diabetes group was reduced to the mild diabetes range (severe-to-mild subgroup), while the mean BG in five mice from the mild diabetes group was reduced to the normal BG range (mild-to-normal subgroup) (Fig. 1c). When including only embryos from dams that maintained severe hyperglycaemia throughout pregnancy, fetal BG levels mirrored maternal BG levels (Fig. 1d). Fetal growth showed a distinct, inverted U-shaped pattern relative to the severity of hyperglycaemia. Embryos from the mice in the mild diabetes group were significantly heavier than those from mice in the non-diabetes and severe diabetes groups, and longer than those from mice in the severe diabetes group (Fig. 1e, f), whereas embryos from the severe diabetes group had the lowest weight among the groups (Fig. 1e). The BG, BW and BL for embryos from dams in the severe-to-mild subgroup were comparable to those for embryos from dams in the non-diabetes and mild diabetes groups (ESM Fig. 1c–e). The IUGR observed in embryos from the severe diabetes cohort was therefore driven entirely by dams that remained severely diabetic throughout pregnancy. In contrast, recovery in mild diabetes dams was not associated with changes in intrauterine growth or the metabolic environment (data not shown), and these embryos were therefore not excluded from the fetal anthropometric analysis. Placental weights were similar across all groups, suggesting that hyperglycaemia did not induce significant placental damage that would affect intrauterine growth (ESM Fig. 1f). Furthermore, there was no significant correlation between fetal and placental weight in all groups combined (ESM Fig. 1g).

Taken together, these results reveal a bimodal effect of maternal diabetes on fetal growth, with mild hyperglycaemia during pregnancy leading to macrosomia, and severe hyperglycaemia causing significant fetal growth restriction.

### Fetal insulin secretion mediates the bimodal growth response and is regulated by beta cell area and function

**Fig. 2:**
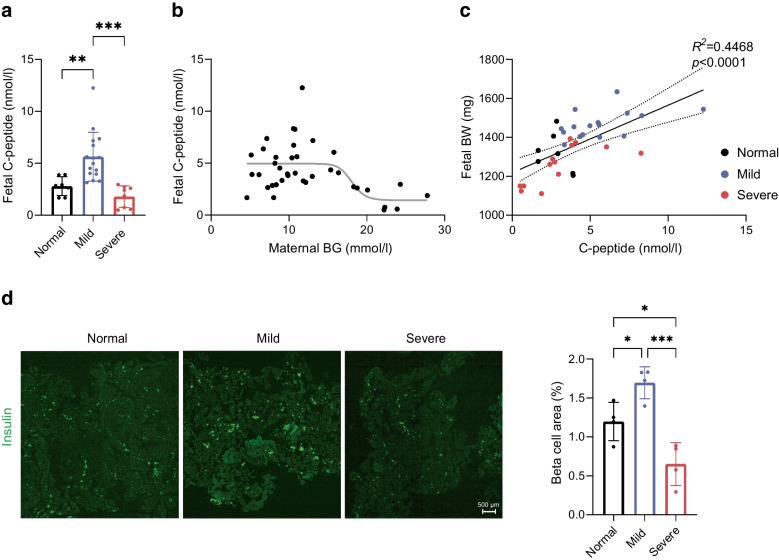
Fetal insulin secretion mediates the bimodal growth response and is regulated by beta cell area. (**a**) Fetal plasma C-peptide concentrations at E18.5 in embryos from dams in the non-diabetes (normal; *n*=7), mild diabetes (*n*=16) and severe diabetes (*n*=8) groups. Five litters from dams with a BG decrease from the severe to the mild diabetes range were excluded. Some plasma samples were not available due to technical limitations. (**b**) Exponential decay curve of fetal C-peptide vs maternal BG. (**c**) Correlation between fetal plasma C-peptide levels and fetal BW. Each point represents the mean litter value. Normal, non-diabetes group. (**d**) Representative immunofluorescence images of fetal pancreatic sections stained for insulin (green) from mice in the non-diabetes (normal; *n*=4), mild diabetes (*n*=4) or severe diabetes (*n*=4) groups. Scale bar, 500 μm. Quantification of beta cell area expressed as a percentage of total pancreatic area is presented for the three groups. Each point represents an individual litter, quantified from analysis of 4–5 sections per litter. Data are presented as mean ± SD (**a**, **d**) or mean only (**b**, **c**), and analysed by two-way ANOVA (**a**, **d**) or linear regression with Pearson correlation (**c**); *p<0.05, **p<0.01, ***p<0.001

Fetal plasma C-peptide levels, a stable marker of insulin secretion, precisely mirrored the inverted U-shaped growth pattern, with levels being highest in the mild diabetes group and significantly lower in the severe diabetes group (Fig. 2a). This link was reinforced by the subgroup analysis: embryos from dams in the severe-to-mild subgroup had significantly higher C-peptide levels than those from dams with permanent severe diabetes, and similar C-peptide levels to embryos from dams in the non-diabetes and mild diabetes groups (ESM Fig. 2a), paralleling the growth pattern of embryos in the corresponding subgroups (ESM Fig. 1c). We demonstrated a significant decline in fetal C-peptide levels using an exponential decay curve at maternal BG levels >16.7 mmol/l (Fig. 2b) and at fetal BG levels >11.1 mmol/l (ESM Fig. 2b), suggesting that these are biologically meaningful transition points in the bimodal insulin response.

The association between fetal C-peptide and intrauterine growth is further emphasised by the finding of a positive correlation between C-peptide levels and both fetal BW and BL (Fig. 2c and ESM Fig. 2c). To uncover the cellular mechanisms that drive the bimodal fetal insulin response, we investigated two key parameters: pancreatic beta cell area and function.

First, we quantified fetal beta cell area by measuring the insulin-positive area relative to the total pancreatic area in tissue sections. The results closely paralleled our in vivo C-peptide measurements. Embryos from the mild diabetes group showed a significant expansion of beta cell area compared with the other groups. In contrast, embryos exposed to severe maternal diabetes had a markedly reduced beta cell area compared with the other groups (Fig. 2d), indicating a smaller volume of insulin-producing cells.

**Fig. 3:**
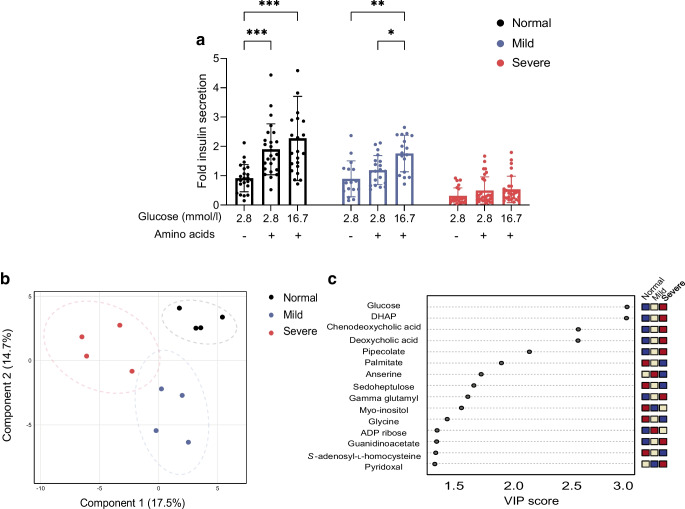
Fetal insulin secretion is regulated by beta cell function. (**a**) GSIS in isolated fetal islets (non-diabetes [normal], *n*=4; mild diabetes, *n*=5; severe diabetes, *n*=5) incubated in 2.8 mmol/l glucose, 2.8 mmol/l glucose plus AA or 16.7 mmol/l glucose plus AA. In total, 5–7 replicates were performed per condition, with nine islets per replicate. Fold change values in insulin secretion were normalised to values obtained in response to 2.8 mmol/l glucose without AA in the non-diabetes group. Data are presented as mean ± SD. (**b**) Partial least squares discriminant analysis of fetal plasma metabolomics data from the non-diabetes (normal; black circles; *n*=4), mild diabetes (blue circles; *n*=4) and severe diabetes (red circles; *n*=4) groups. Each point represents the mean of a litter. (**c**) VIP score plot displaying the score of top metabolites that can be used to discriminate between embryos from dams in the non-diabetes (normal), mild and severe diabetes groups. Data analysed by two-way ANOVA; *p<0.05, **p<0.01, ***p<0.001

Then, we assessed beta cell function using ex vivo insulin secretion assays on isolated fetal islets to evaluate their response to nutrients. As expected, fetal islets from mice in the non-diabetes group displayed the characteristic immature phenotype, responding to AA stimulation regardless of glucose concentration (Fig. 3a). In contrast, fetal islets from the mild diabetes group showed a significant secretory response to high glucose compared with low glucose, with no additional response to AA, resembling a mature beta cell pattern. Fetal islets from the severe diabetes group were functionally impaired, exhibiting a severely blunted insulin secretory response to both glucose and AA stimulation, indicative of cellular exhaustion or damage (Fig. 3a).

Finally, we sought to identify metabolic determinants associated with the bimodal insulin dynamics. Using partial least squares discriminant analysis (Fig. 3b) of fetal plasma samples, we found that the metabolites mainly driving the separation between the non-diabetes, mild and severe diabetes groups were related to glucose and glycolysis (Fig. 3c). This motivated use of a glucose-focused intervention (i.e. a KD) as an appropriate approach to potentially mitigate the fetal growth abnormalities in diabetes during pregnancy.

In summary, our findings suggest a dual mechanism of islet-derived insulin secretion. Mild maternal hyperglycaemia increased beta cell area and enhanced GSIS, which together drove the hyperinsulinaemia that led to fetal overgrowth. Conversely, severe maternal hyperglycaemia appeared to reduce beta cell mass and cause profound functional exhaustion, resulting in insulin deficiency and subsequently IUGR.

### KD normalises fetal growth in mild diabetes but not in severe diabetes

Next, we tested whether nutritional intervention to control maternal hyperglycaemia can mitigate diabetes sequelae. To this end, we introduced a KD immediately after mating, at a mean age of 10.0 ± 3.2 weeks.

**Fig. 4:**
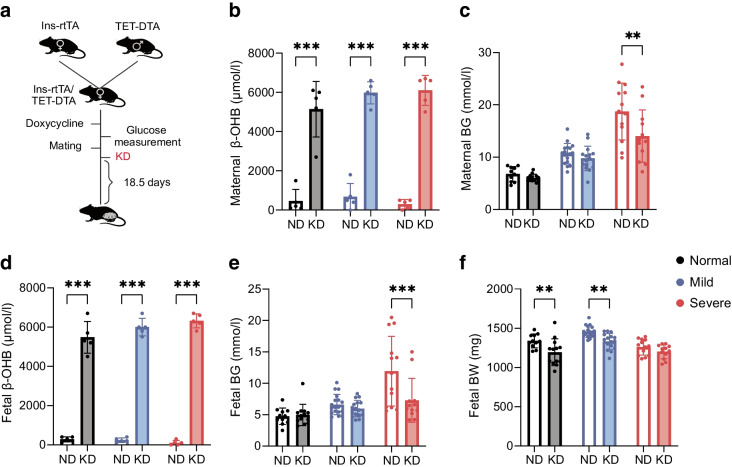
Ketogenic diet (KD) normalises fetal growth in mild diabetes but not in severe diabetes. (**a**) Schematic experimental representation of preconception diabetes induced in Insulin-rtTA;TET-DTA mice, and KD intervention. Created in BioRender. Helman, A. (2026) https://BioRender.com/3p1xwhx. (**b**) Maternal plasma β-hydroxybutyrate (β-OHB) in the non-diabetes (normal), mild and severe diabetes groups under a normal diet (ND) or KD (*n*=5 for all conditions), and (**c**) BG concentrations in the non-diabetes (normal), mild and severe diabetes groups under an ND (*n*=11, *n*=18 and *n*=13, respectively) or KD (*n*=13, *n*=16 and *n*=12, respectively). BG data are missing for *n*=1 dam in the non-diabetes group under ND conditions. (**d**–**f**) Fetal plasma β-OHB (**d**), BG (**e**) and BW (**f**) in the non-diabetes (normal), mild and severe diabetes groups under the ND and the KD. Each point represents the mean litter value. Data are means ± SD, analysed by two-way ANOVA with Tukey’s post hoc test; **p<0.01, ***p<0.001

A total of 41 dams aged 14.2 ± 3.5 weeks at delivery were divided into three KD-fed groups based on mean BG after doxycycline administration and before KD initiation: non-diabetes (‘normal’; *n*=13), mild diabetes (*n*=16) and severe diabetes (*n*=12) (Fig. 4a). Plasma levels of β-hydroxybutyrate, a primary ketone body produced by ketosis, were highly elevated in all KD-fed dams (Fig. 4b), confirming the expected metabolic shift towards ketone production. The glycaemic differences between the groups were maintained on the KD both before pregnancy (ESM Fig. 3a) and during pregnancy (ESM Fig. 3b). However, the KD significantly reduced maternal BG in the severe diabetes group compared with their ND-fed counterparts (Fig. 4c). Maternal BW (measured at E18.5) was similar across the three groups and comparable to maternal BW under the ND (ESM Fig. 3c).

The KD exerted a profound yet divergent influence on fetal outcomes. Fetal plasma β-hydroxybutyrate levels were tenfold higher in the KD-fed group than in the corresponding ND-fed control mice (Fig. 4d), mimicking the levels in the corresponding dams (Fig. 4b). Fetal BG mirrored the maternal glycaemic pattern, decreasing only in the severe diabetes group (Fig. 4e). The KD reduced fetal BW in the non-diabetes and mild diabetes groups but had no effect in the severe diabetes group (Fig. 4f). Notably, in the mild diabetes group, the KD restored fetal BW to the ND range. In both the non-diabetes and mild diabetes groups, these weight changes were not accompanied by changes in fetal BG. Similarly, fetal BG reduction in the severe diabetes group was not associated with a change in BW (Fig. 4e, f), suggesting an uncoupling of the embryonic growth phenotype from fetal BG. We cannot exclude the possibility that the placenta may contribute to the intrauterine growth improvement observed in the mild diabetes group, as placentas in this group were heavier under the KD than under the ND (ESM Fig. 3d). These intrauterine growth effects were independent of litter size, which remained similar across all groups and diets (ESM Fig. 3e).

**Fig. 5:**
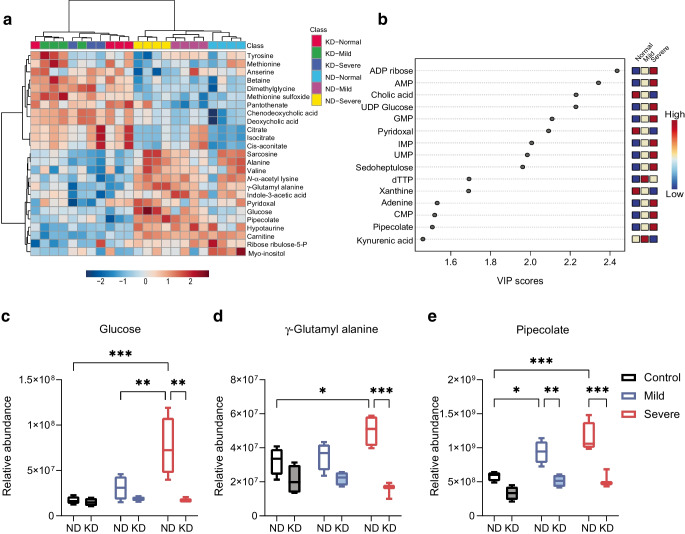
Ketogenic diet (KD) erases metabolic distinctions between diabetes groups. (**a**) Heatmap for metabolites in the non-diabetes (normal), mild and severe diabetes groups under the normal diet (ND) and KD conditions. (**b**) VIP score plot displaying the top metabolites that can be used to discriminate between embryos from dams in the non-diabetes (normal), mild and severe diabetes groups under the KD. (**c**–**e**) Relative abundance of selected metabolites (glucose [**c**], γ-glutamyl alanine [**d**] and pipecolate [**e**]) in the plasma of embryos from dams in the non-diabetes (normal), mild and severe diabetes groups fed the ND or KD. ADP-ribose, adenosine diphosphate ribose; AMP, adenosine monophosphate; CMP, cytidine monophosphate; dTTP, deoxythymidine triphosphate; GMP, guanosine monophosphate; IMP, imidazole propionate; UMP, uridine monophosphate. Box plots in (**c**–**e**) show the median (horizontal line), IQR (box), and minimum/maximum values (whiskers). Data analysed by two-way ANOVA with Tukey’s post hoc test; *p<0.05, **p<0.01, ***p<0.001

To explore the uncoupling of embryonic growth and glucose, we profiled embryonic plasma metabolites across the diabetes groups under the ND and the KD. We observed that, unlike the embryos from dams receiving the ND, those from dams receiving the KD were no longer separable based on their diabetes group (Fig. 5a, b), suggesting that the diet overrides the diabetes-related metabolic profile. Indeed, the KD neutralised differences in key metabolites such as glucose, which was most distinguishable between the diabetes groups under the ND (Fig. 5c), as well as γ-glutamyl alanine and pipecolate (Fig. 5d, e), and myo-inositol (an insulin mimetic [19]) and adenosine diphosphate ribose (ADP-ribose; ESM Fig. 4). However, no single metabolite or pathway fully explained how the diet overrides glucose as the primary determinant of outcomes. Assessment of mTORC1-sensed AA (leucine, arginine and methionine) [20–22] showed only that methionine was elevated in the mild diabetes KD-fed group, suggesting a limited overall contribution of AA to this pathway (ESM Fig. 4). In summary, a KD during pregnancy effectively prevented fetal overgrowth caused by mild maternal diabetes, but was insufficient to correct IUGR in the severe diabetes group. Additionally, because the KD impaired fetal growth in healthy, normoglycaemic pregnancies (Fig. 4f), this nutritional perturbation can potentially have both beneficial and harmful consequences, depending on the mother’s diabetic status.

### KD partially rescued fetal beta cell function and insulin levels in diabetes during pregnancy

To elucidate the cellular mechanism underlying the effects of the KD on fetal growth and glycaemic control, we investigated its effects on insulin secretion, beta cell area and beta cell function in the embryos.

**Fig. 6:**
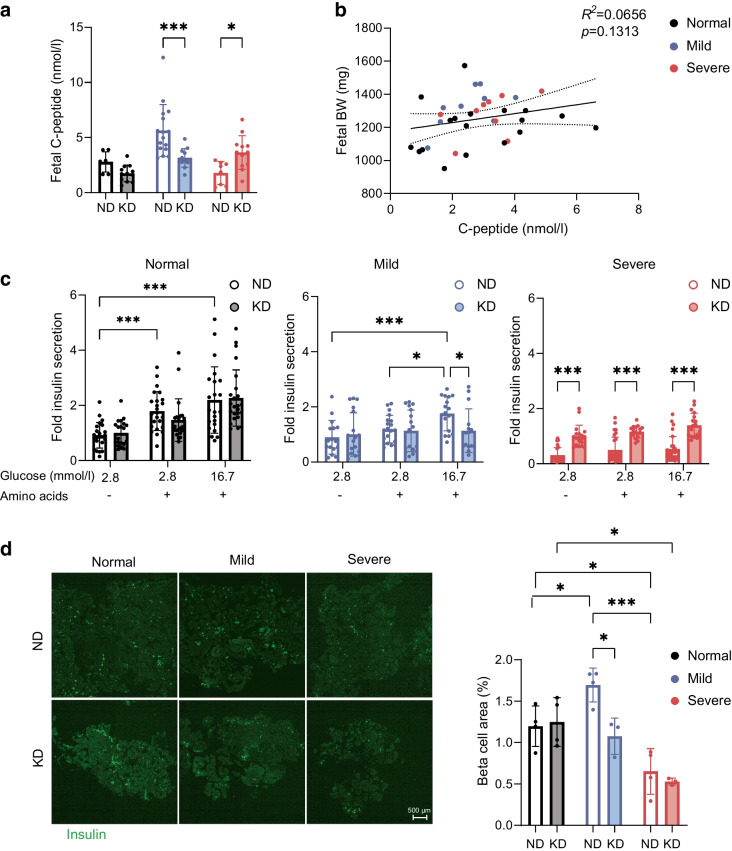
Ketogenic diet (KD) partially rescued fetal beta cell function and insulin levels in diabetes during pregnancy. (**a**) Fetal plasma C-peptide concentrations at E18.5 in the non-diabetes (normal), mild diabetes and severe diabetes groups under the normal diet (ND) (*n*=7, *n*=16 and *n*=8, respectively) or the KD (*n*=13, *n*=16 and *n*=12, respectively). (**b**) Correlation between fetal plasma C-peptide and BW. Each point represents the mean litter value. (**c**) Effect of maternal diet (ND vs KD) on GSIS in isolated fetal islets of the non-diabetes (normal) and diabetes groups incubated with 2.8 mmol/l glucose, 2.8 mmol/l glucose plus AA or 16.7 mmol/l glucose plus AA. In total, 5–7 replicates were performed per condition, with nine islets per replicate. For each diet group, fold change values in insulin secretion were normalised to values obtained for the non-diabetes group in response to 2.8 mmol/l glucose without AA. (**d**) Representative immunofluorescence images of fetal pancreatic sections stained for insulin (green) for the non-diabetes (normal), mild and severe diabetes groups under ND and KD conditions (*n*=4 per group, except for mild and severe diabetes groups under the KD condition, for which only *n*=3 data points presented due to technical error). Scale bar, 500 μm. Quantification of beta cell area expressed as a percentage of total pancreatic area is presented for the three groups across both diets. Each point represents an individual litter, quantified from the analysis of 4–5 sections per litter. The representative images shown for mice in the ND group are identical to those presented in Fig. 2d. Data are presented as mean ± SD (**a**, **c**, **d**) or mean only (**b**). Data in (**a**, **c**, **d**) were analysed by two-way ANOVA with Tukey’s post hoc test; *p<0.05, ***p<0.001. Data in (**b**) were analysed using linear regression with Pearson correlation

The KD exerted opposing effects on fetal C-peptide levels in the two diabetic groups, reducing elevated C-peptide levels in the mild diabetes group and increasing suppressed C-peptide levels in the severe diabetes group compared with their ND-fed counterparts (Fig. 6a). This resulted in similar C-peptide levels between the mild and severe diabetic groups under the KD. Similar to the dissociation between BG and fetal BW (Fig. 4e, f), the KD also disrupted the positive correlation between fetal C-peptide levels and fetal growth (Fig. 6b and ESM Fig. 5a) that was observed under the ND (Fig. 2c and ESM Fig. 2c).

At the cellular level, we performed functional and histochemical studies of fetal islets ex vivo. Overall, the KD did not affect islet-induced insulin secretion or beta cell area in the non-diabetes group (Fig. 6c, d). However, in islets from the mild diabetes group, the KD abolished the glucose responsiveness observed under the ND (Fig. 6c), equalising insulin secretion between AA and glucose stimulations (Fig. 6c). This functional change was associated with a reduction in mean beta cell area compared with its ND counterpart (Fig. 6d). In contrast, in islets from the severe diabetes group, the KD significantly increased insulin secretion in response to all AA and glucose stimulations (Fig. 6c). Although the KD improved insulin secretion both in vivo and ex vivo in the severe diabetes group, this functional recovery was not associated with an improvement in beta cell mass, as beta cell area remained low (Fig. 6d).

In summary, the KD reduced insulin secretion in the mild diabetes group by decreasing both beta cell glucose responsiveness and expansion, whereas it improved beta cell responsiveness in the severe diabetes group without affecting beta cell mass. In addition, the KD had a direct effect on fetal growth, mediated by insulin-independent factors, thereby uncoupling fetal insulin levels from intrauterine growth.

### mTORC1 and MafA expression correlate with changes in fetal beta cell proliferation and maturation

**Fig. 7:**
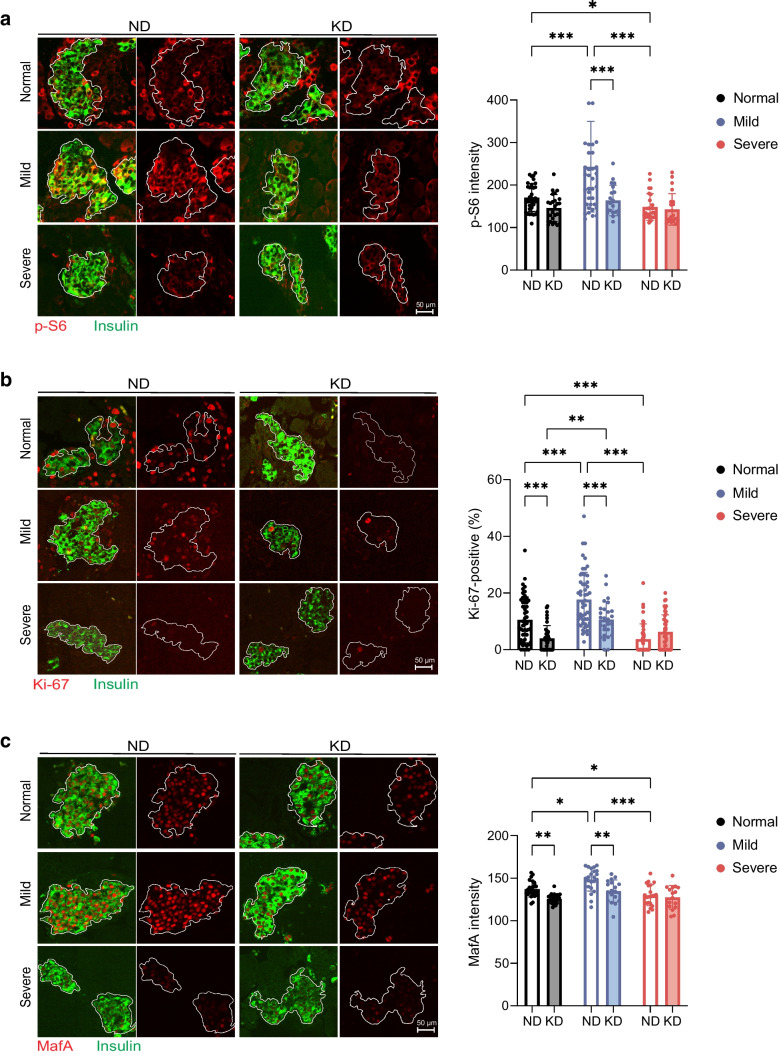
mTORC1 and MafA expression correlate with changes in fetal beta cell proliferation and maturation. (**a**–**c**) Representative immunofluorescence images showing insulin (green) co-stained (red) with p-S6 (**a**), Ki-67 (**b**) or MafA (**c**) in fetal pancreases at E18.5 from the non-diabetes (normal), mild and severe diabetes groups under normal diet (ND) or ketogenic diet (KD) conditions (*n*=4 per group). Scale bars, 50 μm. Quantification of mean fluorescence intensity (for p-S6 and MafA) or percentage of positive cells (for Ki-67) is also shown. Each point represents an individual islet, quantified across three sections from each litter. Data are means ± SD, analysed by two-way ANOVA; *p<0.05, **p<0.01, ***p<0.001

To uncover the molecular drivers of the observed changes in fetal beta cells, we investigated the mTORC1 pathway, which governs beta cell proliferation and maturation, by staining for its downstream target, p-S6. For mice receiving the ND, fetal islets from the mild diabetes group showed strong mTORC1 activation, as indicated by high p-S6 intensity (Fig. 7a). This high p-S6 intensity aligned with increased beta cell proliferation, as shown by greater beta cell area (Fig. 2d) and a higher percentage of Ki-67-positive beta cells (Fig. 7b). In contrast, mTORC1 activity was low in islets obtained from embryos of dams with severe diabetes, consistent with reduced beta cell area (Fig. 2d) and a lower percentage of Ki-67-positive beta cells (Fig. 7b). The KD completely normalised mTORC1 activation in fetal islets from the mild diabetes group, reducing p-S6 staining to levels comparable with those from the non-diabetes group under both diets (Fig. 7a), consistent with the reversal of beta cell hyperplasia (Fig. 6d) and the reduced beta cell Ki-67 expression in this group (Fig. 7b). However, in the severe diabetes group, the KD had no effect on mTORC1 activity in fetal islets, consistent with the absence of change in beta cell area (Fig. 6d) and Ki-67 expression (Fig. 7b). In addition to beta cell proliferation (as reflected by the percentage of Ki-67-positive beta cells, Fig. 7b), beta cell area was also affected by beta cell size. Mean beta cell size was larger for the mild diabetes group than for the non-diabetes group under the ND, and was reduced under the KD (ESM Fig. 5b), consistent with the diverse patterns of beta cell area (Fig. 6d).

Next, to understand differences in glucose responsiveness, we assessed expression of MafA, a key transcription factor in beta cell functional maturation. In line with the ex vivo pattern of GSIS (Fig. 3a), MafA intensity was significantly upregulated in the fetal islets of the mild diabetes group fed the ND, confirming premature intrauterine beta cell maturation (Fig. 7c). In contrast, under ND conditions, MafA expression was reduced in the severe diabetes group compared with both the non-diabetes and mild diabetes groups (Fig. 7c), consistent with beta cell functional exhaustion (Fig. 3a). The KD reversed the elevated MafA levels, significantly lowering MafA intensity in both the non-diabetes and mild diabetes groups compared with its ND counterpart, restoring it to a level commensurate with a more immature state (Fig. 7c). In contrast, the KD did not change MafA intensity in the severe diabetes group (Fig. 7c).

Taken together, these findings provide a molecular basis for the observed beta cell phenotypes. Mild maternal hyperglycaemia increases fetal beta cell mass (in terms of both beta cell proliferation and size) via the mTORC1 pathway while simultaneously inducing premature functional maturation, as evidenced by MafA upregulation. The KD effectively reversed these changes by suppressing both mTORC1 signalling and MafA expression, thereby normalising fetal beta cell mass and function.

## Discussion

This study examined the metabolic impact of maternal diabetes on fetal growth and beta cell maturation in vivo and ex vivo under various glycaemic conditions and dietary regimens. A mouse model was used because severe diabetes cannot be ethically studied in humans, and a KD is not recommended during pregnancy. The results showed a bimodal effect of maternal glucose on fetal intrauterine growth, and found that a KD can mitigate some but not all of these pathological changes through insulin-dependent and insulin-independent mechanisms.

The importance of precise glycaemic control for intrauterine growth was emphasised by the fact that some dams underwent spontaneous partial recovery from diabetes, a phenomenon that has been previously reported in this model [17, 18]. In our cohort, the BG in dams that recovered from severe to mild diabetes was sufficiently decreased to shift the intrauterine glycaemic environment from insulin-suppressive to insulin-stimulatory, significantly improving fetal C-peptide levels and growth. In contrast, dams whose BG level decreased from that of the mild diabetes group to that of the non-diabetes group did not show similar improvements in fetal outcomes, probably because their BG, while lower, remained at the high end of the normal range.

Given the critical role of maternal hyperglycaemia in intrauterine growth, we evaluated use of a nutritional intervention (KD) to modify the glycaemic environment of the dams and embryos. KDs have been shown to improve glycaemic control, insulin secretion and insulin sensitivity in non-pregnant adults with obesity and type 2 diabetes [23–26]. We hypothesised that shifting the primary maternal energy source away from carbohydrates would reshape the fetal metabolic environment and mitigate diabetes-induced growth impairment. Indeed, in the mild diabetes group, the KD reduced fetal glucose exposure, thereby halting the pathological overstimulation of fetal beta cells. Conversely, in the severe diabetes group, the diet reduced the hyperglycaemic burden, allowing fetal beta cells to partially recover function.

The KD exerted insulin-independent growth restriction, as evidenced by reduced BW in embryos from dams in the non-diabetes group and a disrupted C-peptide/BW correlation. This restriction, combined with reduced insulin, corrected macrosomia in the mild diabetes group but was counterbalanced by elevated insulin in the severe diabetes group, preventing growth rescue. While a KD is currently discouraged during pregnancy due to risk of embryo development and neural tube defects [1, 13–15], previous studies were confounded by insufficient folic acid [13, 14]. As mild carbohydrate restriction is well-tolerated [1, 12], and a KD during pregnancy successfully reshapes fetal beta cell function in embryos of dams with mild diabetes, use of a nutritionally modified KD during pregnancy warrants investigation as a potential therapy for macrosomia.

Our study confirms that fetal beta cells in a healthy environment display a characteristically immature phenotype, with insulin secretion poorly stimulated by glucose [3, 5–8, 27]. However, in embryos exposed to mild maternal hyperglycaemia, we observed a switch toward a premature maturation phenotype in isolated islets ex vivo. This finding, consistent with changes to plasma C-peptide levels in vivo, extends previous reports by showing that in utero hyperglycaemia reprogrammes the fetal beta cell [3, 6–8].

As the mTORC1 pathway is the nutrient-sensing pathway that dictates the postnatal shift from AA to glucose responsiveness [5, 28], we evaluated its role in fetal beta cell development. Mild maternal hyperglycaemia creates a unique metabolic environment that enhances mTORC1 expression in islets, correlating with two key outcomes: premature functional maturation via transcription factors such as MafA [29], and beta cell expansion, as indicated by increased area, elevated beta cell size and an increased number of Ki-67-positive cells [10, 30, 31]. Consequently, mTORC1 acts as the molecular hub linking mild hyperglycaemia to both hyperfunctional and hyperplastic fetal beta cells. Conversely, severe hyperglycaemia impairs beta cell function and reduces beta cell fitness, reflecting glucotoxicity rather than stimulation [18]. This bimodal severity-dependent response underscores the need to precisely define hyperglycaemia ranges in murine diabetes studies.

In conclusion, our findings refine the Pedersen hypothesis by demonstrating that fetal growth outcomes directly mirror the state of the fetal beta cell, which may range from hyperfunctional to damaged. While IUGR in severe diabetes is often attributed to placental microangiopathy [32, 33] and impaired nutrient transport [34], our results reveal that primary failure of the fetal endocrine pancreas, induced by severe hyperglycaemia, is an additional mechanism restricting growth. This finding underscores that the fetal beta cell is an active participant whose fate, and that of the fetus, depends on precise maternal glycaemic management. Given the metabolic benefits of a KD observed here, further studies should evaluate use of a balanced KD as a medical nutrition therapy for pregnancies in women with diabetes.

## Supplementary Information

Below is the link to the electronic supplementary material.

ESM (PDF 627 KB)

## Data Availability

The data generated and analysed during the current study are available after publication, with no restriction, from the corresponding author on reasonable request.

## Abbreviations

AA: Amino acids
BG: Blood glucose
BL: Body length
BW: Body weight
GSIS: Glucose-stimulated insulin secretion
IUGR: Intrauterine growth restriction
KD: Ketogenic diet
mTORC1: Mechanistic target of rapamycin complex 1
ND: Normal diet
p-S6: Phosphorylated ribosomal protein S6
