# Supplementary material for: Fetal growth is driven by a bimodal fetal beta cell response and reshaped by a ketogenic diet in a mouse model of maternal diabetes
Source: Diabetologia. 2026 Jun 9;69(9):2634–48. doi: 10.1007/s00125-026-06771-w (PMC13424211; doi:10.1007/s00125-026-06771-w)
Supplement: Supplementary file 1 — ESM (PDF 627 KB) [file 125_2026_6771_MOESM1_ESM.pdf]

ESM methods

Mice

Animal studies were conducted in accordance with the guidelines of the Authority for Biological and Biomedical Models at the Hebrew University of Jerusalem, Israel. The study was approved by the Ethics Committee of the Faculty of Agriculture, Food and the Environment, The Hebrew University of Jerusalem (MD-22-168544). Mice were housed in a Specific Pathogen-Free (SPF) facility on a 12-hour light/12-hour dark cycle and were given either a normal diet (ND) or a ketogenic diet (KD) ad libitum. ICR mice were purchased from Envigo RMS, Israel. We used the insulin-rtTA; TET-DTA mice model. In this model, the rat insulin2 promoter drives the expression of reverse tetracycline-dependent transactivator (rtTA) in β-cells [1, 2]. These mice were crossed with mice expressing the diphtheria toxin A subunit (DTA) under the control of an rtTA-responsive promoter (TET). The transgenic mice were genotyped by PCR of tail tips using primers for insulin-rtTA and TET-DTA as previously reported [1]. To activate diphtheria toxin and induce apoptosis in β-cells, doxycycline (Tamar Laboratory Supplies, DB0889, Israel) was administered in the drinking water (2 mg/mL doxycycline, 4 % w/v sucrose) for 7 days.

Insulin and C-peptide measurements

Insulin secretion from fetal islets ex vivo was measured using an Ultra-Sensitive Rat Insulin ELISA kit, and plasma C-peptide levels were determined using a Mouse C-peptide ELISA kit, according to the manufacturer's instructions (Crystal Chem, 90050, IL, USA). The minimum detection sensitivity in the insulin and C-peptide assays was 8.6 pmol/L and 0.03 nmol/L, respectively, and both assays had intra- and inter-assay precision <10%. We measured plasma C-peptide rather than insulin because hemolysis, which was common during blood sampling from the embryos, reduces insulin levels but does not affect C-peptide levels [3].

Immunohistochemistry

Pancreatic tissue slices were used for immunohistochemical staining to detect insulin, phosphorylated ribosomal protein S6 (p-S6), MAFA, and Ki67. Primary antibodies included guinea pig anti-insulin (Agilent IR002; 1:5), rabbit anti-phospho-S6 Ribosomal Protein (Ser240/244) (Cell Signaling; 5364L; 1:300), Ki67 (Abcam; ab16667; 1:300), and rabbit anti-MAFA (Cell Signaling; #79737; 1:300). Secondary antibodies conjugated to Alexa Fluor 488 or Cy5 were used for visualization. Slides were mounted using Invitrogen Fluoromount-G mounting medium, and coverslips were applied. Images were acquired using an Andor BC43 confocal microscope. Subsequent image analysis was performed using QuPath version 5.1.

Measurement of β-Hydroxybutyrate

Maternal and fetal plasma β-hydroxybutyrate (βOHB) concentrations were quantified using the FreeStyle Optium Neo ketone monitoring system. Briefly, plasma samples were thawed, and a 1.5 µL aliquot was applied directly to the application zone of FreeStyle Optium β-ketone test strips (Abbot, 7869001, IL, USA). The meter measures βOHB via an enzymatic electrochemical reaction (β-hydroxybutyrate dehydrogenase), providing a readout in mmol/L with a detection range of 0.0 to 8.0 mmol/L.

LC-MS polar metabolite analysis

1 µL of each sample was injected into a ZIC-pHILIC 150 x 2.1 mm (5 µm particle size) column (EMD Millipore) operated on a Vanquish™ Flex UHPLC system (Thermo Fisher Scientific). Chromatographic separation was achieved using the following gradient: Buffer A = 95% acetonitrile + 5% 20 mM ammonium carbonate, 0.1% ammonium hydroxide in water; Buffer B = 95% 20 mM ammonium carbonate, 0.1% ammonium hydroxide in water + 5% acetonitrile. Gradient conditions used were: 0-17.5 min: linear gradient from 16.6% to 75% B; 17.5-18.0 min: linear gradient from 75% to 85% B; 18-20 min: hold at 85% B; 20-20.5 min: from 85% to 16.6% B; 20.5-24 min: hold at 16.6% B at 0.150 mL/min flow rate. The column oven and auto sampler tray were held at 25°C and 4°C, respectively. MS data acquisition was performed using a QExactive

benchtop Orbitrap mass spectrometer equipped with an Ion Max source and a heated electrospray ionization (HESI) IIprobe (ThermoFisher Scientific) with polarity switching. Tune file parameters were: spray voltage = 3.5 kV, capillary temperature = 320°C, S-lens radio frequency (RF) = 50, auxiliary (aux) gas temperature= 350°C. Two scans were used: full scans in both positive and negative ionization mode in a range of m/z = 70-1,000 m/z, resolution = 70,000, AGC target = 1 × 106, and max IT = 40 ms from 0-17.5 minutes.

Metabolomics data analysis

Polar metabolites were relatively quantified while referencing an in-house library of chemical standards and using Trace Finder 4.1 (ThermoFisher Scientific, Waltham, MA, USA) with a 5 parts per million (ppm) mass tolerance. Pooled samples and fractional dilutions were prepared as quality controls and injected at the beginning and end of each run. In addition, pooled samples were interspersed throughout the run to control for technical drift in signal quality and to assess the coefficient of variation (CV) for each metabolite. Data normalisations were performed in two steps: (i) Integrated peak area signal from internal standards added to extraction buffers were mean-centered (for every standard, peak area was divided by the mean peak area of the set) and averaged across samples; samples were divided by the resulting factor, thus normalising for any technical variability due to MS signal fluctuation or pipetting and sample injection errors (usually within 10% variability). (ii) Normalisation for biological material was based on detected polar metabolites as follows: CV values (based on pooled sample reinjections) and coefficient of determination [R-squared, (based on linear dilutions of pooled sample)] were calculated per metabolite. Metabolites with <30% CV and >0.95 RSQ were mean-centered and averaged across samples. Metabolite peak areas were then divided by the resulting factor (biological normaliser), thus accounting for any global shift in metabolite amounts due to differences in biological material.

References

1. Nir T, Melton DA, Dor Y (2007) Recovery from diabetes in mice by β cell regeneration. Journal of Clinical Investigation 117(9):2553–2561. https://doi.org/10.1172/JCI32959

2. Furth-Lavi J, Hija A, Tornovsky-Babeay S, et al (2022) Glycemic control releases regenerative potential of pancreatic beta cells blocked by severe hyperglycemia. Cell Rep 41(9). https://doi.org/10.1016/j.celrep.2022.111719

3. Zornitzki T, Blaychfeld-Magnazi M, Knobler H, Ulman M (2015) The Effect of Phlebotomy-Induced Hemolysis on Insulin Level Determination. Endocrine Practice 21(10):1093–1097. https://doi.org/10.4158/EP15698.

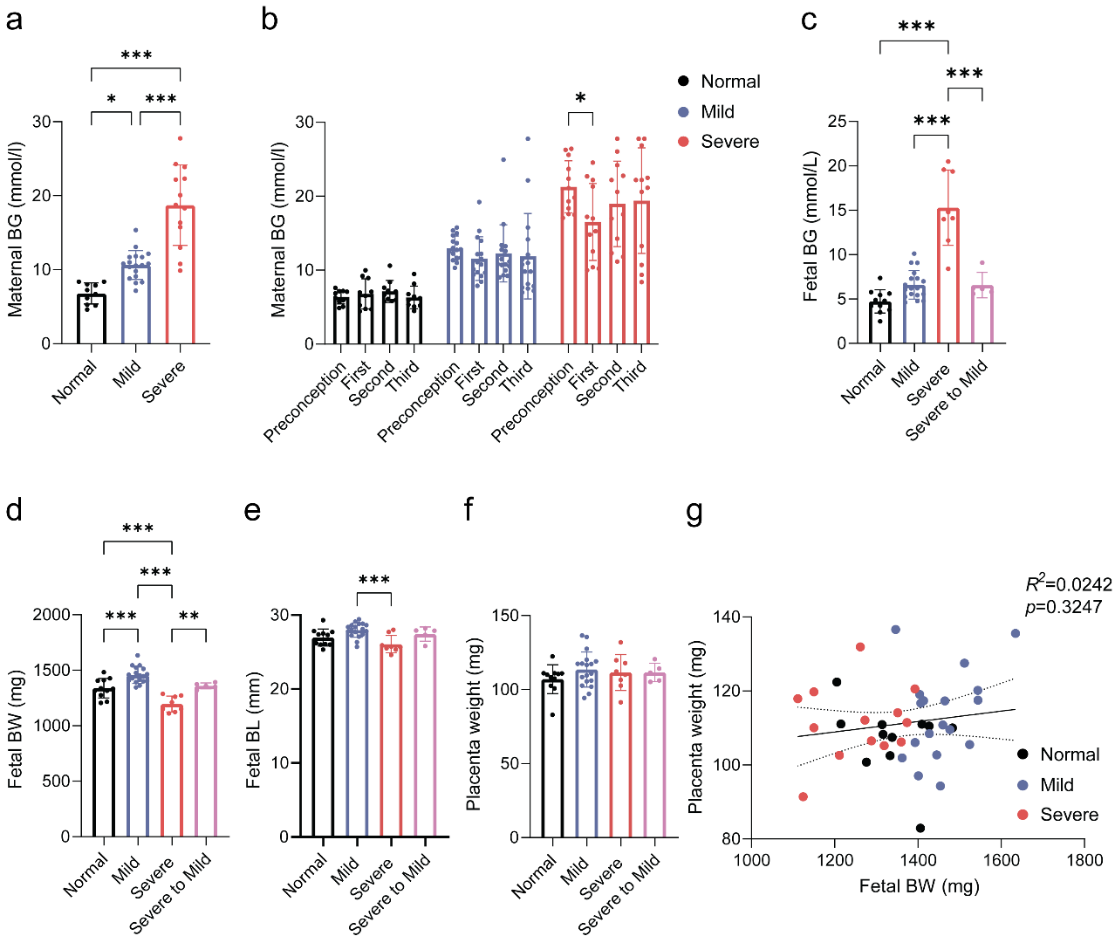

ESM Fig. 1. Maternal diabetes severity dictates fetal growth in a bimodal manner. (**a**) Mean blood glucose (BG) of dams during pregnancy based on twice-weekly measurements in the normal (n=11), mild diabetes (n=18), and severe diabetes (n=13) groups. (**b**) Maternal BG levels before and during pregnancy, dissected by gestational trimesters. (**c**) Fetal BG, (**d**) body weight (BW), and (**e**) body length (BL) in the normal, mild diabetes, and the two severe diabetes subgroups, *i.e.*, permanent severe diabetes and severe-to mild diabetes. (**f**) Placenta weight of normal, mild diabetes and the two subgroups of severe diabetes. (**g**) Correlation between placenta weight and fetal BW. Each point represents the mean litter value. All data are shown as mean ± SD; analyzed by one-way ANOVA and two-way ANOVA with multiple comparisons. *p<0.05, **p<0.01, ***p<0.001.

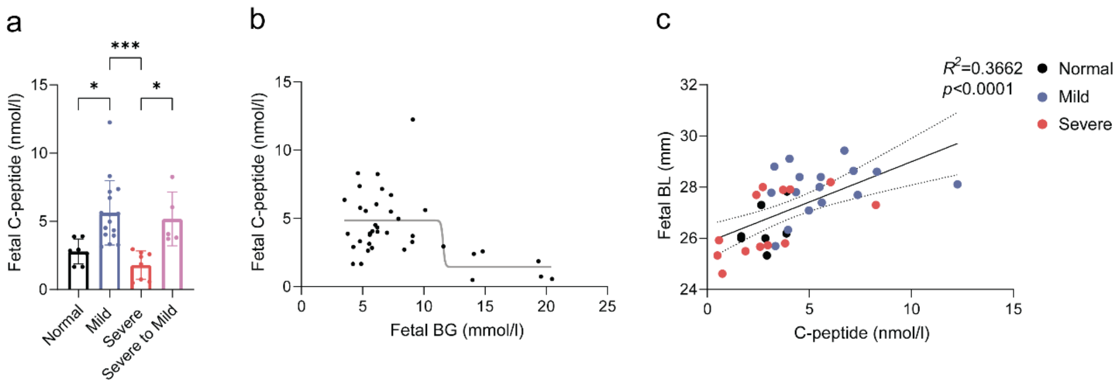

ESM Fig. 2. Fetal insulin secretion mediates the bimodal growth response. (**a**) Fetal plasma C-peptide concentrations at E18.5 in normal (n=7), mild diabetes (n=16), and the two severe diabetes subgroups, *i.e.,* permanent severe diabetes (n=8) and severe-to mild diabetes (n=5) groups. Some plasma samples were not available due to technical limitations. (**b**) Exponential decay curve of fetal C-peptide *vis-à-vis* fetal blood glucose (BG). (**c**) Correlation between fetal C-peptide levels and fetal body length (BL). Each point represents the mean litter value. Data are shown as mean ± SD; analyzed by two-way ANOVA. *p<0.05, ***p<0.001.

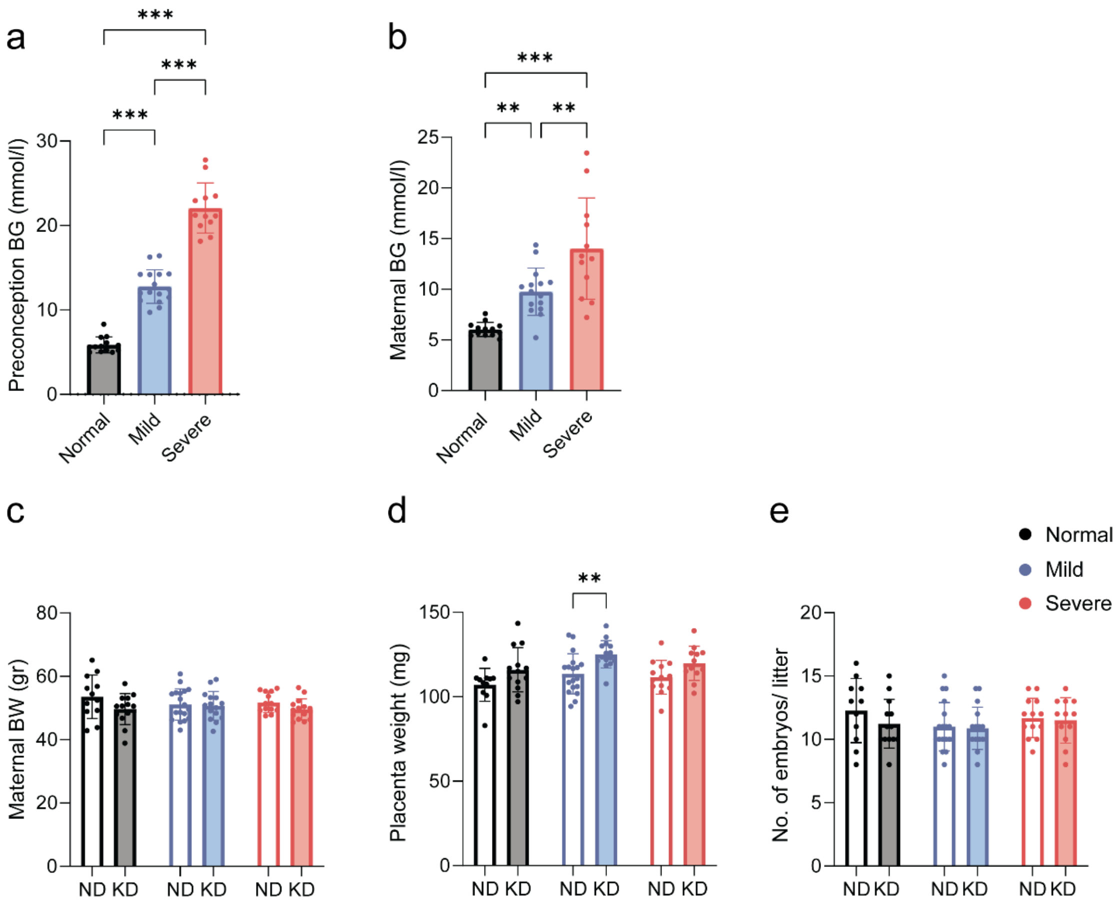

ESM Fig. 3. Ketogenic diet normalises fetal growth in mild diabetes but not in severe diabetes. Maternal blood glucose (BG) (**a**) before and (**b**) during pregnancy in normal (n=13), mild (n=16), and severe (n=12) diabetes groups under ketogenic diet (KD). (**c**) Maternal body weight (BW) at delivery was similar across all groups and diets. (**d**) Placenta weight at E18.5 in normal, mild, and severe diabetes groups under normal diet (ND; n=11, 18, 13 respectively) or ketogenic diet (KD; n=13, 14, 12 respectively). Each point represents the mean litter value. Placenta weight data are missing for two dams of the mild diabetes group under KD. (**e**) Litter size between the three groups of normal, mild and severe diabetes under both diets. ***p*<0.01, ****p*<0.001.

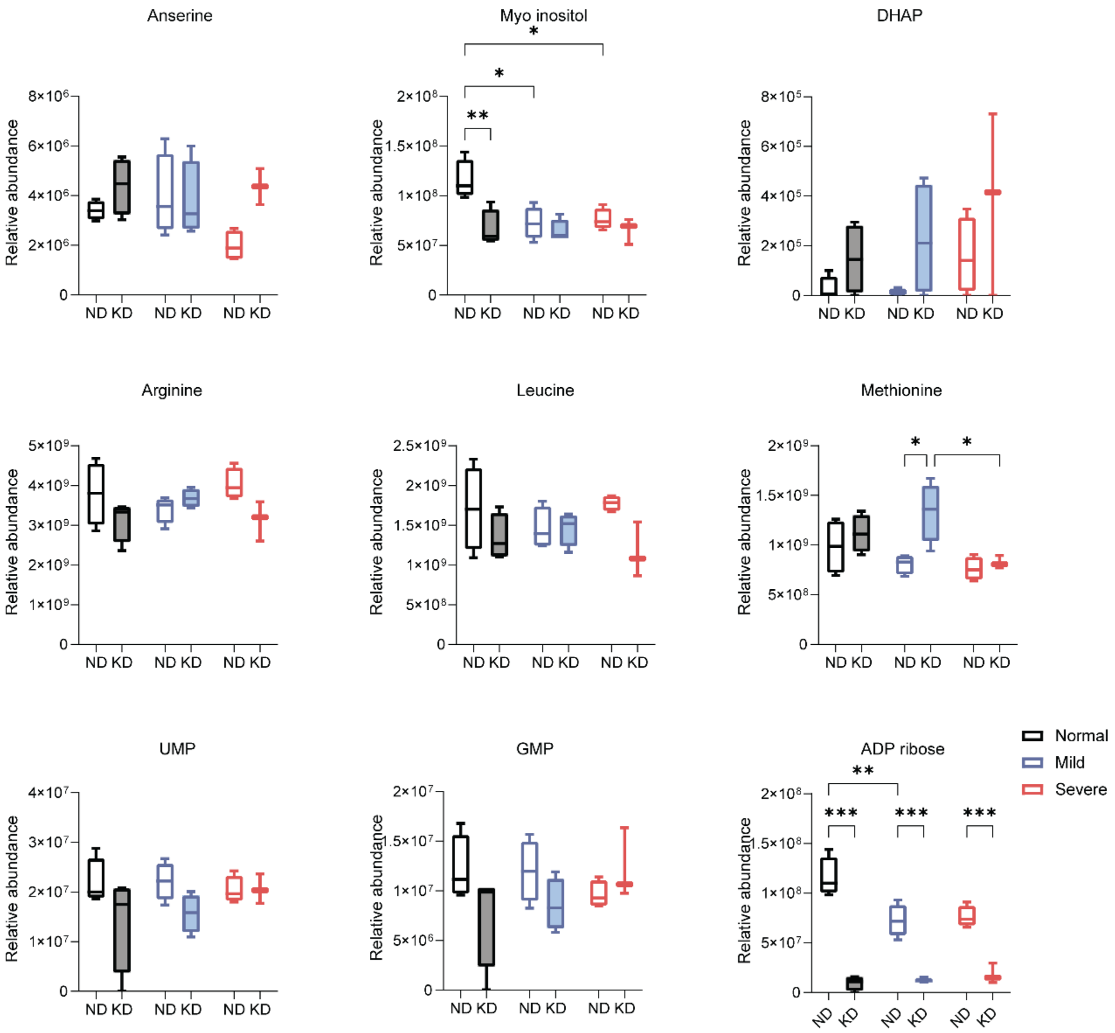

ESM Fig. 4. Ketogenic diet erases metabolic distinctions between diabetes groups. Differential abundance of selected metabolites in the plasma of embryos from normal, mild, and severe diabetic dams fed a normal diet (ND) or a ketogenic diet (KD). Dihydroxyacetone phosphate (DHAP); Uridine Monophosphate (UMP); Guanosine monophosphate (GMP); Adenosine diphosphate ribose (ADP ribose). Each point represents the mean litter value. Data are shown as mean ± SD; analyzed by two-way ANOVA. *p<0.05, **p<0.01, ***p<0.001.

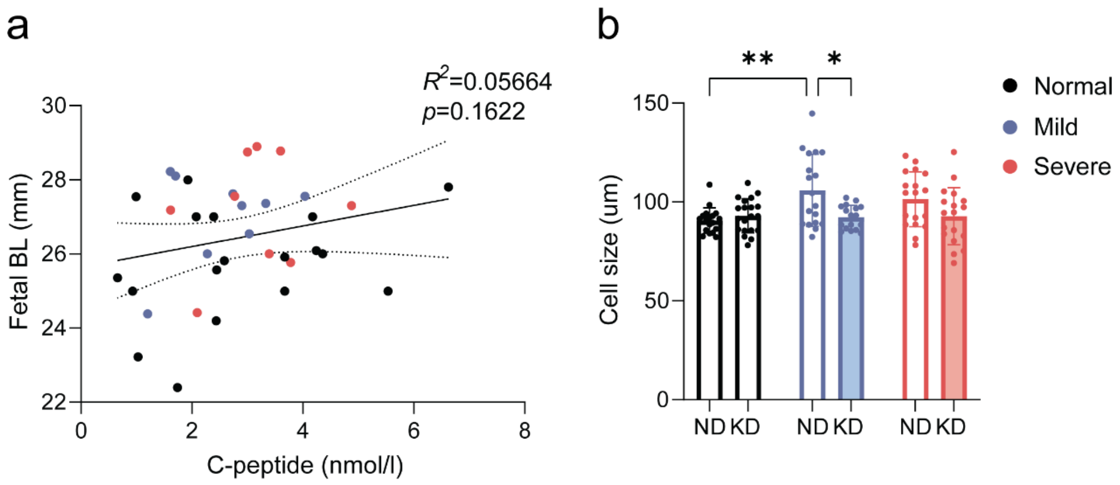

ESM Fig. 5. Ketogenic diet partially rescued fetal β-cell function and insulin levels in diabetes during pregnancy. (**a**) Correlation between fetal C-peptide and body length (BL). Each point represents the mean litter value. (**b**) Mean β-cell size in pancreas sections from embryos of normal, mild, and severe diabetes groups under both diets. Cell size was quantified across three sections from each litter. Data are shown as mean ± SD; analyzed by two-way ANOVA. *$p<0.05$, **$p<0.01$.
